# A Rare Case of Prosthetic Joint Infection Caused by Group D *Salmonella*

**DOI:** 10.1155/crdi/7477436

**Published:** 2026-02-17

**Authors:** Anna Barbiero, Lucia Graziani, Matteo Piccica, Francesco Raspanti, Luigi Zanna, Marco Mugnaini, Letizia Attala, Massimo Antonio Di Pietro

**Affiliations:** ^1^ Dipartimento di Medicina Sperimentale e Clinica, Università Degli Studi di Firenze, Florence, Italy, unifi.it; ^2^ SOC Malattie Infettive, Ospedale Santa Maria Annunziata, Azienda USL Toscana Centro, Bagno a Ripoli, Florence, Italy, uslcentro.toscana.it; ^3^ SOC Ortopedia e Traumatologia, Ospedale Santa Maria Annunziata, Azienda USL Toscana Centro, Bagno a Ripoli, Florence, Italy, uslcentro.toscana.it

## Abstract

Prosthetic joint infections (PJIs) are gaining growing attention as a healthcare issue. *Salmonella* spp. is a rare cause of PJI, mostly involving hip arthroplasty rather than knee arthroplasty. Therefore, clinical experience on the management of PJI involving knee arthroplasties caused by *Salmonella* spp. is scant. We report a case of knee PJI caused by Group D *Salmonella*, successfully treated with a two‐stage revision surgery and appropriate antibiotic therapy, based on antimicrobial susceptibility of the cultural isolate. Such challenging cases evidence that clinical success is achievable through appropriate medical and surgical management, combined with a strong collaboration between orthopedics and infectious disease specialists.

## 1. Introduction

The number of total joint arthroplasties (TJAs) performed annually has increased dramatically in last decades, due to general aging of population and excellent functional outcome [1]. Between 2001 and 2016, total knee arthroplasty (TKA) procedures increased with an average of 6.6% each year in Italy, with an upward trend predicted for the coming years [2].

Prosthetic joint infections (PJIs) are among the most devastating complications following TJAs, with important repercussions on quality of life and mental health, as well as economic burden on healthcare systems [3, 4]. It represents one of the main complications following TJAs with incidence rates of 1%–3%. While gram‐positive bacteria are the most frequently involved pathogens in PJIs, gram‐negative bacteria cause a minority of reported PJIs [5]. *Salmonella* spp. is a rare cause of PJI, being reported in literature in 0.3% of cases; it is more frequently associated with total hip arthroplasty (THA) PJIs rather than TKA [6, 7]. According to available literature, the experience on clinical management and related outcomes in case of TKA caused by *Salmonella* spp. is currently limited [7–10]. This work reports a case of TKA infection caused by a Group D *Salmonella* successfully treated with a two‐stage revision surgery and appropriate antimicrobial therapy.

## 2. Case Presentation

The case involves a 68‐year‐old woman from Romania. Her clinical history included hypothyroidism, diabetes, asthma, Wegner’s granulomatosis in chronic corticosteroid therapy, and allergy to ciprofloxacin, penicillin, and ampicillin (she reported anaphylactic shock after amoxicillin/clavulanate administration).

In April 2021, she underwent a TKA surgery in Romania due to osteoarthritis with severe instability of the left knee. After surgery, the patient complained persistent pain and swelling of the knee. Pain worsened 6 months after surgery, and she noticed the appearance of a fistula secerning purulent material, without any systemic sings of infection. The patient presented to our attention at the end of November 2021. At clinical examination, the left knee was swelled, red, and tender on palpation, motion range was decreased, and a fistula was present on the lateral side. X‐rays showed a well‐fixed implant (Figure 1).

FIGURE 1Left knee X‐ray before explantation, at the first clinical evaluation in our center. (a) Laterolateral projection; (b) anteroposterior projection.

**Figure:**
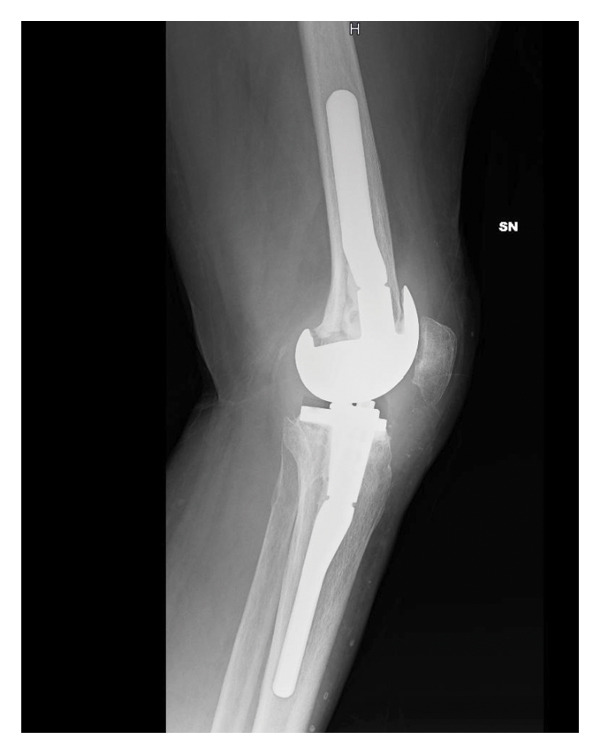
(a)

**Figure:**
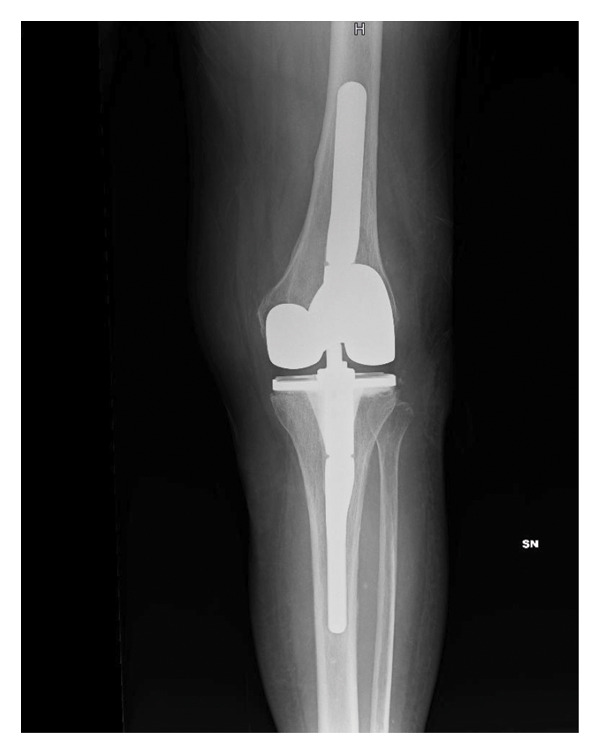
(b)

An arthrocentesis was performed, showing synovial leukocytosis (> 3000 cells/mm^3^) with 70% of polymorphonuclear leukocytes and positive alpha‐defensin and leukocyte esterase. The cultural exam was positive for Group D *Salmonella* (antimicrobial susceptibility profile is shown in Table 1). Due to the presence of a sinus tract, the multidisciplinary “orthoinfective” team addressed the patient to a two‐stage approach. On 6 December 2021, she underwent the first‐stage procedure, with removal of the infected implant, a radical synovectomy, and debridement of all infected soft tissues and bones. A temporary articulated‐spacer plus intramedullary antibiotic cement‐coated fiches containing gentamicin was implanted (Figure 2). Of note, in vitro activity of aminoglycosides does not necessarily correlate with complete in vivo effectiveness in case of *Salmonella* spp. infection due to the possibility of intracellular infection; therefore, independently of MIC values, these isolates are always reported as resistant in our laboratory. However, since aminoglycosides MIC values were below the lowest MIC threshold in this case (Table 1), it was considered clinically acceptable to use a gentamicin‐based cemented spacer combined with appropriate targeted systemic antibiotic therapy.

**TABLE 1:** Antimicrobial susceptibility testing of Group D *Salmonella* isolated from the patients’ synovial fluid.

Antibiotic	Susceptibility result	MIC (μg/mL)
Amikacin	R	≤ 4
Ampicillin	S	≤ 4
Amoxicillin–clavulanate	S	≤ 2/2
Ceftazidime	S	≤ 1
Ciprofloxacin	R	0.25
Ceftriaxone	S	≤ 1
Cefotaxime	S	≤ 1
Ertapenem	S	≤ 0.25
Cefepime	S	≤ 1
Gentamicin	R	≤ 1
Imipenem	S	≤ 0.25
Levofloxacin	S	≤ 0.5
Meropenem	S	≤ 0.125
Tobramycin	R	≤ 2
Trimethoprim/sulfamethoxazole	S	≤ 1/19
Ceftazidime/avibactam	S	≤ 0.25/4
Piperacillin/tazobactam	S	≤ 4/4
Ceftolozane/tazobactam	S	≤ 0.5/4

*Note:* R = resistant, S = susceptible.

Abbreviation: MIC = minimum inhibitory concentration.

FIGURE 2Left knee X‐ray after implantation of the articulated spacer. (a) Laterolateral projection; (b) anteroposterior projection.

**Figure:**
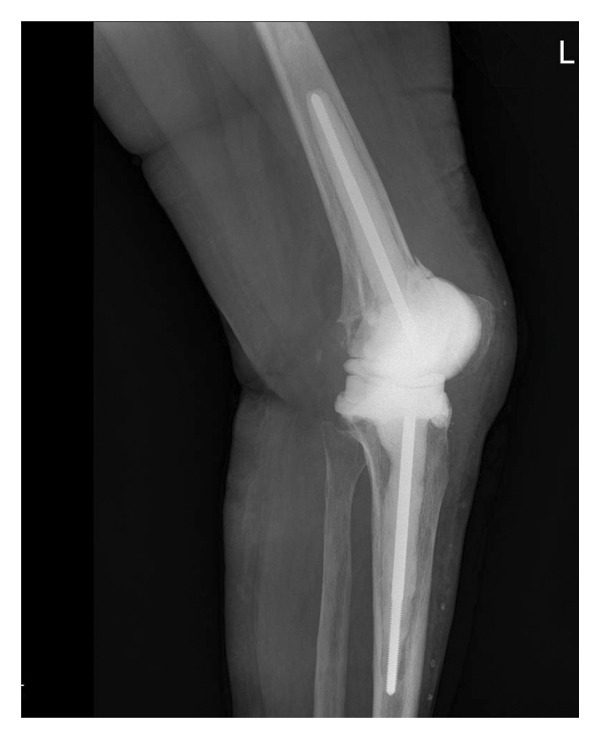
(a)

**Figure:**
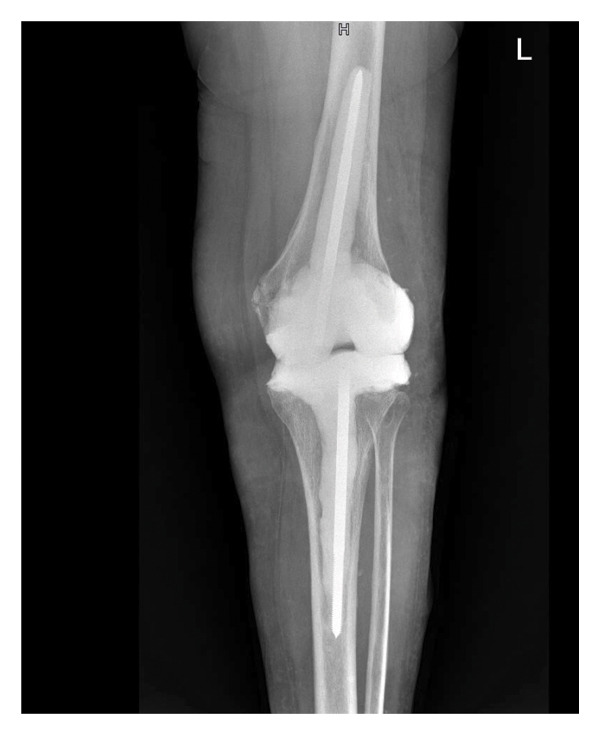
(b)

Group D *Salmonella* grew in 5 out of 5 intraoperative cultures, with an antimicrobial susceptibility profile comparable to the one obtained from synovial culture.

Postoperatively, the patient was treated with trimethoprim/sulfamethoxazole (TMP‐SMX) 160 mg/800 mg per os tid.

To exclude any primary foci of infection, an abdomen ultrasound and a cardiac ultrasound were performed with negative results. Blood culture exams resulted negative.

Two weeks after surgery, due to evidence of a progressive leuko‐neutropenia, TMP‐SMX therapy was discontinued, and treatment with ertapenem (1 gr/day iv) was started, with subsequent recovery of blood cells counts.

After the first‐stage procedure, the patient remained apyretic, and C‐reactive protein, erythrocyte sedimentation rate, and procalcitonin values decreased gradually till normalization. No signs of local infection were observed during the follow‐up.

The antibiotic therapy was administered for 11 weeks, three more than planned, due to slight increase of the inflammation indexes at week 8, which then spontaneously decreased to normal range.

After 2 weeks of “antibiotic holiday,” an arthrocentesis was performed, and the cultural exam resulted negative. The reimplantation surgery was then scheduled for early May 2022; however, due to a bilateral traumatic fracture of the distal radius followed by a pauci‐symptomatic SARS‐CoV‐2 infection, it was postponed to the beginning of July.

In the second‐stage procedure, a radical synovectomy was performed with aggressive debridement of the joint (Supporting Information Figures 1–4). After spacer explantation, lavage with bactericidal solution made of hydrogen peroxide and povidone‐iodine, a constrained condylar knee prosthesis was implanted (Supporting information Figures 5–7).

One out of 7 intraoperative samples resulted positive for *Staphylococcus capitis,* interpreted as a culture contamination consistently with the other 6 negative cultures. Postoperatively, the patient was prescribed with TMP/SMX 160 mg/800 mg per os tid for 4 weeks. The medication was well tolerated in this occasion. Four weeks after surgery, the wound healed, and blood tests were normal. One year after surgery, there were no signs of infection, blood tests were persistently negative, the scar was in good condition, the patient reported no pain when walking with full weight, and the knee had a motion range of 0°–110°. X‐rays showed good integration of the implant (Figure 3).

FIGURE 3Left knee X‐ray 1 year after prothesis reimplantation. (a) Laterolateral projection; (b) anteroposterior projection.

**Figure:**
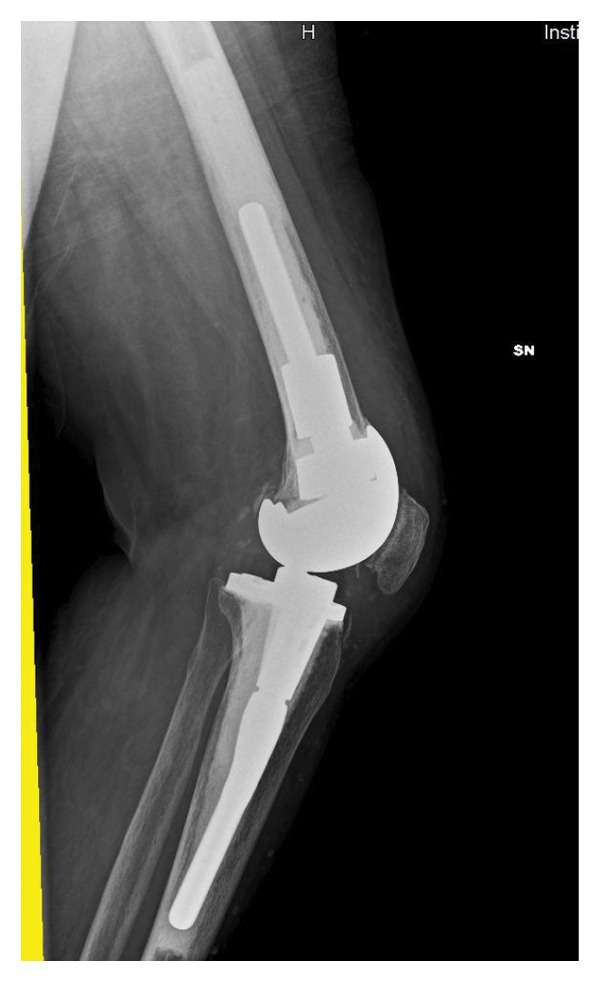
(a)

**Figure:**
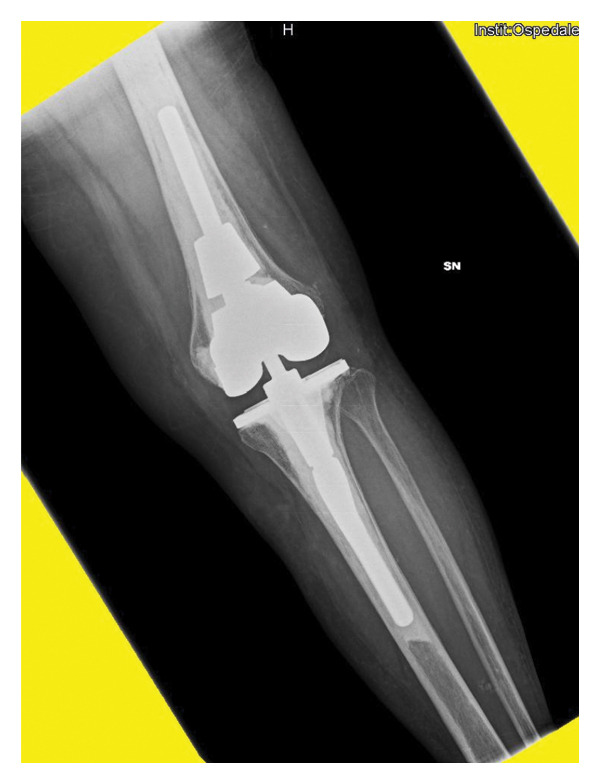
(b)

## 3. Discussion

*Salmonella* spp. infections are frequently associated with gastroenteritis, often self‐limiting in the case of nontyphoid *Salmonella* spp. [10]; between 0.2% and 0.6% of people infected with nontyphoid *Salmonella*, and in 3% of infections with typhoid *Salmonella*, chronic carrier status can follow the acute infection phase. Although rare, hematogenous dissemination and subsequent organ localization are possible, both in acute phases and in chronic carriers. Involvement of the musculoskeletal system may present with reactive arthritis, abscess formation, osteomyelitis, and septic arthritis [11]. PJI caused by *Salmonella* spp. is rare. A review of the literature by Rajgopal et al. documented 32 reported cases of THA and 11 cases of TKA *Salmonella* spp. infection [7]. Afterward, two more cases of TKA *Salmonella* spp. infection have been reported, in 2017 and 2019 [9, 12] (Table 2).

**TABLE 2:** Cases of TKA infection caused by *Salmonella* spp. according to literature review.

Study	Age	Gender	Underlying conditions	*Salmonella* species	Management	Outcome
Rae et al.[13]	67	Female	Rheumatoid arthritis	*Salmonella typhimurium*	Chloramphenicol and amoxicillin, prosthesis retention, chronic suppression with amoxicillin	Chronic suppression
Boland et al. [14]	51	Female	Rheumatoid arthritis	*Salmonella enteritidis*	Multiple drainages with needle aspiration and intravenous ceftriaxone for 4 weeks	Uneventful for 1‐year follow‐up
Madan et al. [15]	75	Female	Rheumatoid arthritis	*S. enteritidis*	Ciprofloxacin for 6 weeks	Recurrence after 15 months, successfully treated with ciprofloxacin for 3 months
Day et al. [16]	55	Male	Type II diabetes mellitus	*S. enteritidis*	Debridement, polyexchange, and retention followed by ceftriaxone for 6 weeks	No recurrence
Musante et al. [17]	63	Female	Gout	*S. typhimurium*	Irrigation and debridement with polyexchange, followed by ceftazidime for 6 weeks	Cured
Miron et al. [18]	75	Male	n.a.	*S. enteritidis*	Debridement and retention, followed by 3 weeks of intravenous ceftriaxone and 3 months of ciprofloxacin	Cured
Kobayashi et al. [19]	71	Female	Rheumatoid arthritis	*S. enteritidis*	Debridement with polyexchange, chronic therapy with ciprofloxacin	Persistent warmth over joints, no other evidence of infection
Oe et al. [20]	61	Male	Rheumatoid arthritis	*S. enteritidis*	Debridement with implant retention, followed by meropenem and levofloxacin for 2 weeks and oral minocycline for 3 months	Cured
Carlile et al. [21]	71	Male	n.a.	*Salmonella choleraesuis*	Two‐stage revision surgery, followed by intravenous. Cefotaxime for 1 week and ciprofloxacin for. weeks	Cured
De la Torre et al. [22]	62	Male	Rheumatoid arthritis	*S. enteritidis*	Two‐stage revision	Recurrence at 9 months
Gupta et al. [6]	n.a.	n.a.	Rheumatoid arthritis, bladder cancer	*S. enteritidis*	TMP‐SMX for 3 months	Failure at 3 months
Sebastian et al. [9]	72	Female	Diabetes mellitus, hypertension, seizure disorder, tubercular meningitis	*S. typhimurium*	Prosthesis removal, debridement, cement spacer application, followed by ciprofloxacin for 6 weeks	Cured (refused second stage for prosthesis reimplantation)
Raviraj et al. [12]	75	Female	None	*Salmonella typhi*	Debridement and polyexchange followed by intravenous ceftriaxone for 2 weeks and ciprofloxacin for 6 weeks	PJI recurrence after 2 years without *Salmonella* spp. identification on knee aspirate

*Note:* n.a. = information not available.

Overall, three out of the 13 reported cases were caused by *S. typhimurium*, 8/13 by *S. enteritidis*, one case by *S. typhi*, and another one by *S. choleraesuis*. It was not possible, in our case, to identify the serotype of the isolated *Salmonella* strain, although the pathogen was identified as belonging to Group D according to the Kauffman–White classification [23]. *S. typhimurium* (Group B) and *S. enteritidis* (Group D) [6] and, in general, nontyphoid *Salmonellae* appear to be a more common cause of PJI than typhoid *Salmonellae* [7]; this may be due to the wider global distribution of the former [24]. In most of the previously reported cases, the infection affected a single joint, whereas bilateral involvement is much rarer.

Patients with thalassemia, systemic lupus erythematosus and any kind of primary or secondary immunosuppression, as confirmed by the literature review reported in Table 2, are at greatest risk for PJI caused by *Salmonella* spp. In these groups, episodes of bacterial translocation from the gastrointestinal tract with dissemination into the blood stream and subsequent focal infection in various anatomical sites, including joints, bone, and prosthetic devices, seem to occur more frequently [3, 6–10, 20, 22]. Indeed, *Salmonella* spp. can invade and survive not only inside macrophages but also inside nonphagocytic cells, including the intestinal epithelium, from which hematogenous seeding can take place [11]. Coherently with other reports in literature, our patient had no gastrointestinal symptoms prior to the PJI, and the coprocultural exams excluded a chronic carrier status [3, 6, 9, 22]. Immunosuppression caused by the chronic corticosteroid intake could have favored hematogenous spread of the pathogen, after a transitory gastrointestinal localization, which then infected the prosthesis, although the origin of the infection in this case remains undetermined [24].

When choosing the most appropriate management of PJI caused by *Salmonella* spp., the property of these microorganisms of forming biofilms must be taken into account; therefore, biofilm‐active antibiotics must be administered, and more invasive and radical surgery with a two‐stage procedure should be preferred to more conservative approaches [11, 25].

With regard to the choice of type of surgical intervention, the patient had been reporting onset of symptoms for about 3 weeks at the time of admission. However, two‐stage surgery is preferable despite the recent onset of symptoms if the prosthesis has been in place for more than 1 month and is recommended in the case of the presence of a fistula, which represents a clear sign of chronic infection [26–29].

In particular, for PJI caused by *Salmonella* spp., an average of 5 years between the first implantation and the onset of infection has been reported, and, despite the onset of symptoms being acute, approaches involving prosthesis retention have been associated with unfavorable outcomes, especially in the case of immunosuppressed patients [6, 22, 30]. These data, combined with the patient’s comorbidities, the presence of a sinus tract and good general status, explain the choice, in our case, of proceeding with a two‐stage intervention. As regarding the choice of performing arthrocentesis prior to reimplantation after a 2‐week “antibiotic holiday,” not all favor obtaining synovial fluid culture to assess sterilization of the joint space [27]. However, given the insidious pathogen, we preferred obtaining synovial fluid cultures due to high clinical concern regarding the presence of persistent infection.

Among the antibiotics of choice in PJI caused by *Salmonella* spp., fluoroquinolones are related to better outcomes due to their excellent bioavailability, high bone penetration, and action on biofilm; other treatment options often include ampicillin, third‐generation cephalosporins, and TMP‐SMX [31, 32].

In our case, the choice of TMP‐SMX was mainly driven by the patient’s allergies and the pathogen’s susceptibility profile, just as the switch to ertapenem was necessary due to the occurrence of worsening leucopenia.

In conclusion, TJAs are expected to increase exponentially over time, together with PJIs. *Salmonella* spp. is an uncommon cause of PJI that needs to be considered in case of infection, especially in the immunocompromised patient and even in the absence of recent gastrointestinal manifestations. Rapid diagnosis, choice of the most appropriate antibiotic therapy and of the most effective and acceptable surgical approach, is the key element to ensure better outcomes.

## Funding

Open access publishing facilitated by Universita degli Studi di Firenze, as part of the Wiley‐CRUI‐CARE agreement.

## Ethics Statement

Ethic committee approval is not required by our institution for case reports. Good Clinical Practice recommendations were followed, in accordance with Helsinki’s Declaration.

## Consent

The patient gave informed consent for the publication of this paper.

## Conflicts of Interest

The authors declare no conflicts of interest.

## Supporting Information

Figure 1 Reimplantation surgery: appearance of the knee preoperatively with marked scars of previous intervention.

Figure 2 Reimplantation surgery: recision following previous surgical scar.

Figure 3 Reimplantation surgery: synovial liquid aspiration.

Figure 4 Reimplantation surgery: articulated spacer exposition.

Figure 5 Reimplantation surgery: articulated spacer removal.

Figure 6 Reimplantation surgery: reimplanted condylar knee prosthesis.

Figure 7 Knee appearance after reimplantation surgery, with postoperative drainage.

## Supporting information


**Supporting Information** Additional supporting information can be found online in the Supporting Information section.

## Data Availability

Data sharing is not applicable to this article as no datasets were generated or analyzed during the current study.
