# Supplementary material for: A Rare Case of Prosthetic Joint Infection Caused by Group D Salmonella
Source: Case Rep Infect Dis. 2026 Feb 17;2026:7477436. doi: 10.1155/crdi/7477436 (PMC12913687; doi:10.1155/crdi/7477436)
Supplement: Supplementary file 1 — Supporting Information Additional supporting information can be found online in the Supporting Information section. [file CRDI-2026-7477436-s001.docx]

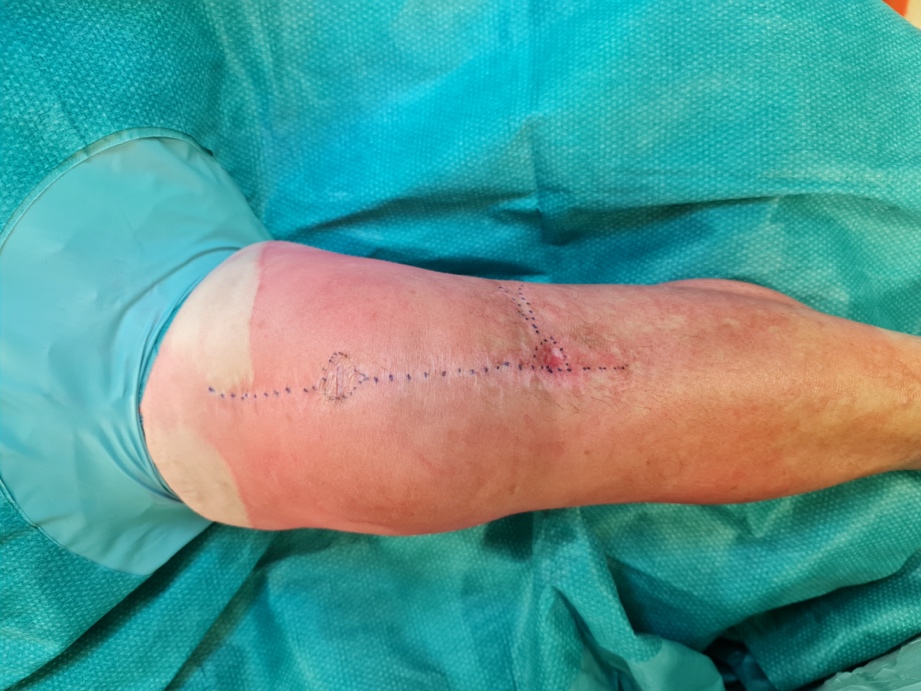


**Fig.1** Re-implantation surgery: appearance of the knee preoperatively with marked scars of previous intervention


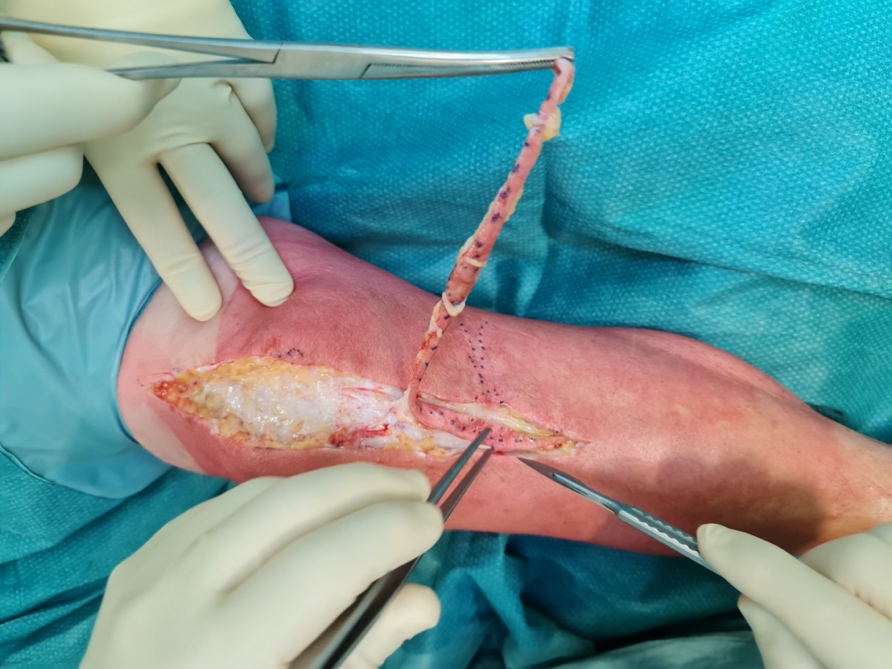


**Fig.2** Re-implantation surgery: recision following previous surgical scar


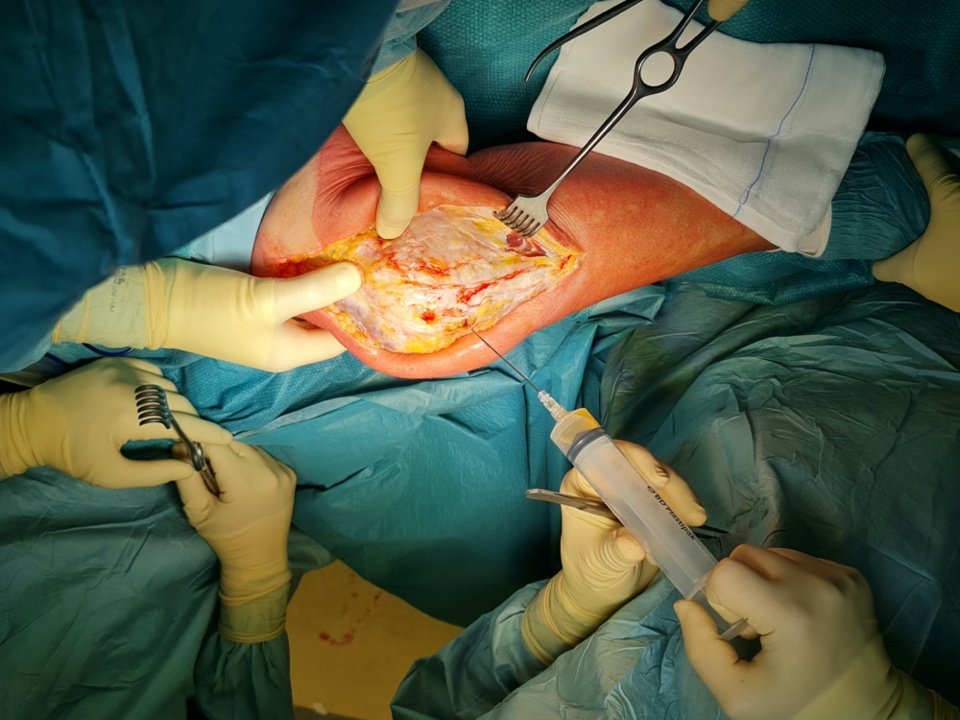


**Fig. 3** Re-implantation surgery: synovial liquid aspiration


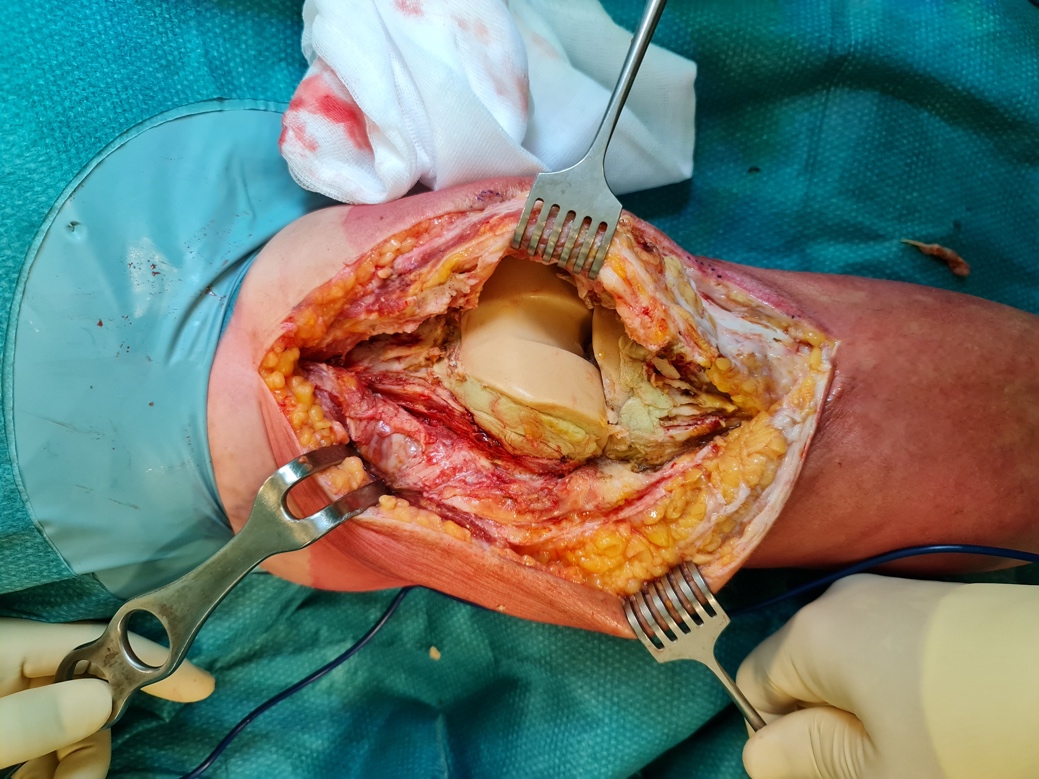


**Fig. 4** Re-implantation surgery: articulated spacer exposition


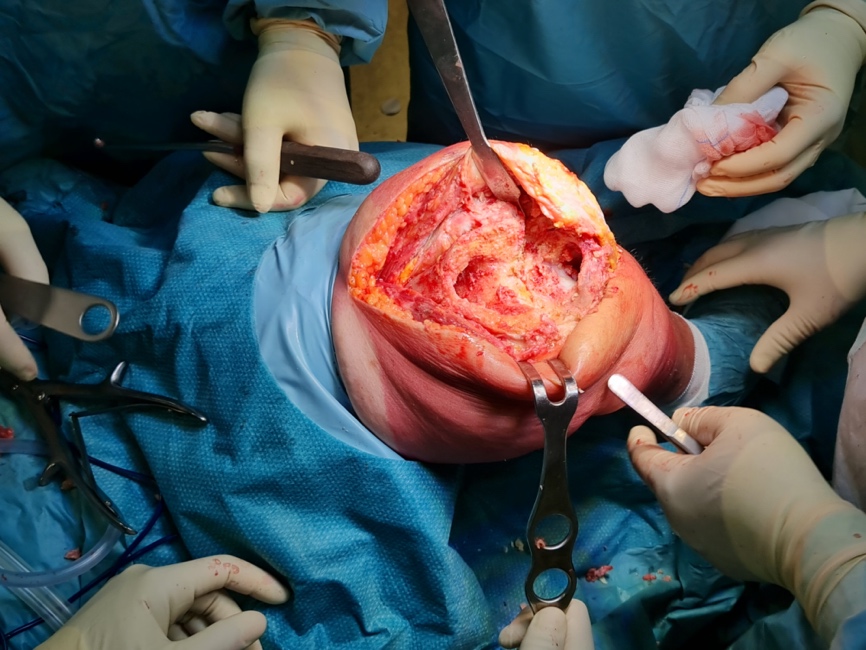


**Fig. 5** Re-implantation surgery: articulated spacer removal


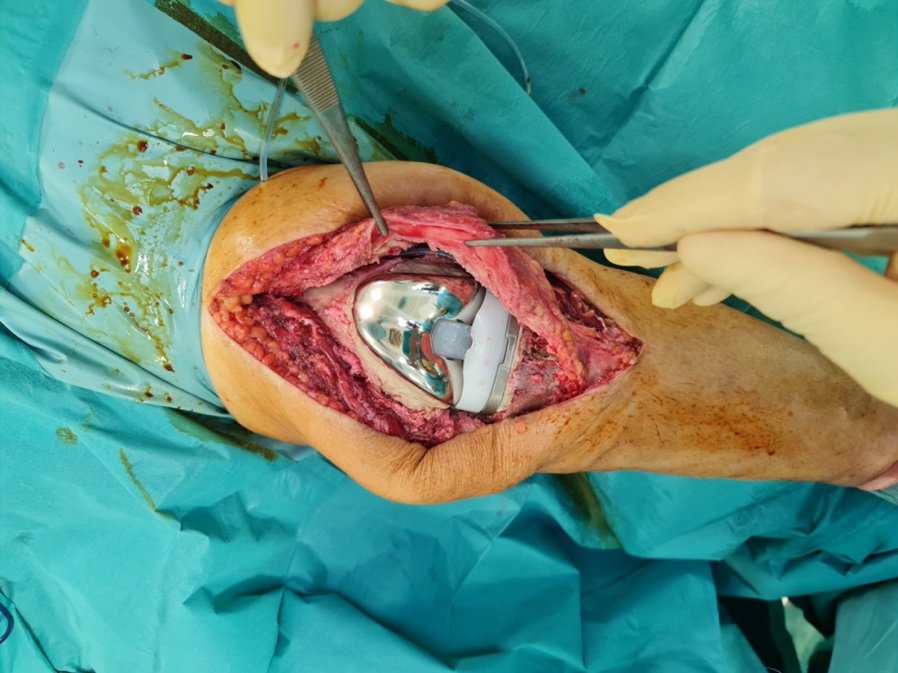


**Fig. 6** Re-implantation surgery: re-implanted condylar knee prosthesis


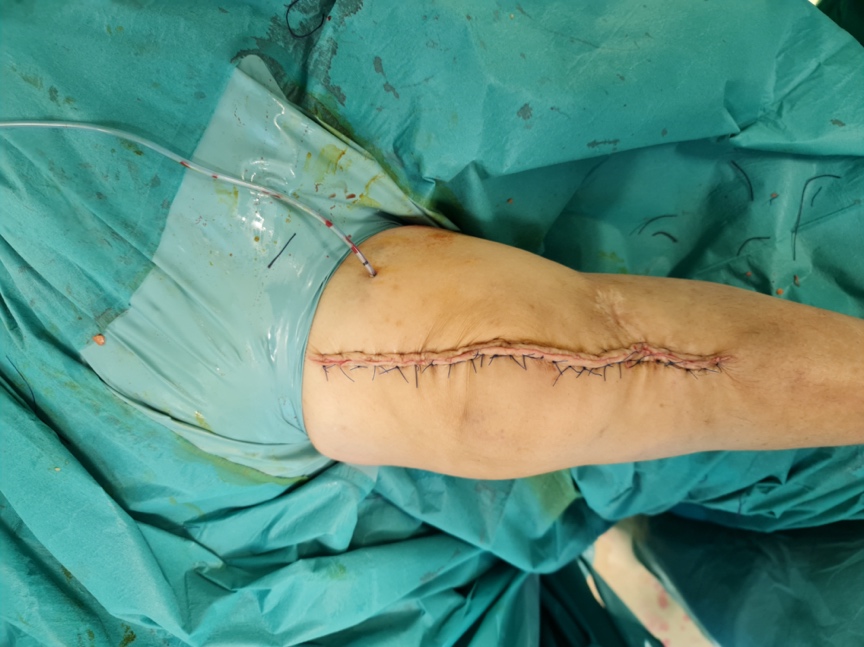


**Fig. 7** Knee appearance after re-implantation surgery, with post-operative drainage
